# Internuclear Ophthalmoplegia Secondary to Cocaine Abuse

**DOI:** 10.1155/2017/2379697

**Published:** 2017-02-06

**Authors:** Richard L. Rabin, Azeem Wasay, Nicolas Biro, Marcelle Morcos

**Affiliations:** ^1^Department of Ophthalmology, Nassau University Medical Center, 2201 Hempstead Turnpike, East Meadow, NY 11554, USA; ^2^Lincoln Medical and Mental Health Center, 234 E. 149th St., Bronx, NY 10451, USA; ^3^Neuro-Ophthalmology, Department of Ophthalmology, Nassau University Medical Center, 2201 Hempstead Turnpike, East Meadow, NY 11554, USA

## Abstract

*Purpose*. To report a case of internuclear ophthalmoplegia (INO) caused by cocaine.* Method*. We report a case of a 54-year-old female who presented with a left INO three days after snorting cocaine, and we review the literature.* Results*. MRI of the brain demonstrated several small abnormal foci in the pons on FLAIR and diffusion weighted imaging consistent with ischemic infarction. The patient's symptoms remained stable throughout her hospitalization. She was sent to a rehabilitation facility and was lost to follow-up.* Conclusion*. In cases of extraocular movement abnormalities, it is important to inquire about recreational drug use.

## 1. Introduction

Internuclear ophthalmoplegia (INO) secondary to cocaine induced ischemic stroke is a rare event with few reported cases in the literature. Diaz-Calderon et al. and Strupp et al. report two similar cases; however, neither is identical to our patient [1, 2]. The pathogenesis of cocaine induced stroke is controversial but likely is secondary to acute hypertension, loss of cerebral vascular autoregulation, cerebral vasospasm, increased platelet activation, and in some cases cardiac thromboembolism [3–6].

## 2. Case Presentation

A 54-year-old heroin user with a history of hypertension presented to the emergency department three days after snorting cocaine. She reported leg weakness, unsteadiness, and diplopia that began one day after using cocaine. She denied using any other substances at that time and denied tobacco use.

**Figure 1:**
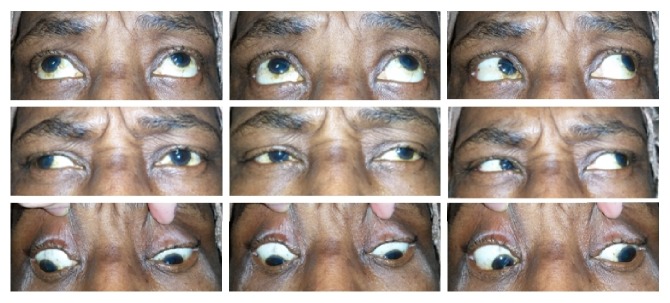
Left INO with absent left eye adduction. Exotropia and left skew deviation are present.

Her blood pressure on presentation was 117/77 with a heart rate of 77 beats per minute. Visual acuity was 20/60 OD and OS. Pinhole acuity was 20/40 OD and 20/60 OS. Autorefraction showed mild myopia. Vision did not improve with refraction. A relative afferent pupillary defect was present in the left eye. Intraocular pressure was 32 OD and 36 OS. Anterior segment exam revealed mild cataracts but was otherwise unremarkable. Fundus exam showed cup-to-disk ratios of 0.6 and 0.85 in the right and left eyes, respectively. Examination of the maculae was unremarkable OU. In primary gaze, the patient had an exotropia and a left hypertropia. Extraocular movements revealed absent left adduction and the presence of right abducting nystagmus with attempted right gaze consistent with a left INO with left skew deviation (Figure 1; see online suppl. video 1, in Supplementary Material available online at https://doi.org/10.1155/2017/2379697).

**Figure 2:**
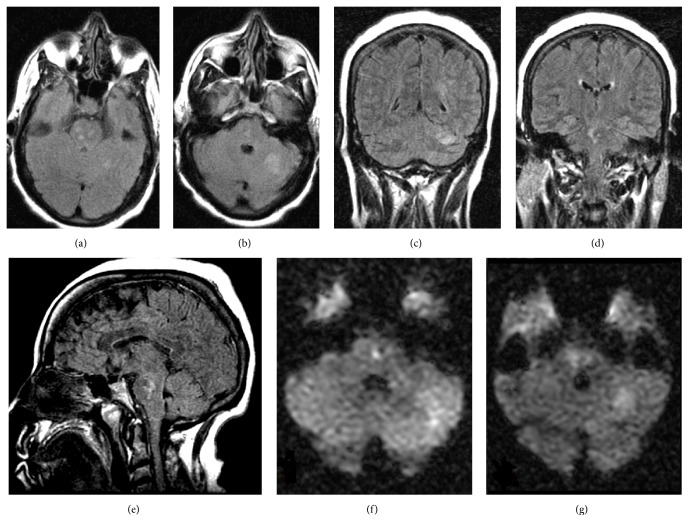
Brain MRI. (a, b, c) Abnormal FLAIR signal involving the left cerebellum. (a, b, d, e) Abnormal FLAIR signal involving several small foci within the pons. (f and g) Diffusion weighted imaging sequence consistent with multiple regions of acute ischemia in the pons and left cerebellum.

OCT of the macula was unremarkable OU. MRI of the brain demonstrated abnormal FLAIR, with diffusion weighted imaging revealing several small foci within the pons bilaterally and the left cerebellum consistent with multiple regions of acute ischemia (Figure 2). MRI showed no evidence of multiple sclerosis. MRA of the head and neck were normal. Transthoracic echocardiogram showed impaired relaxation of the left ventricle and a hyperdynamic left ventricle in systole. Left ventricle ejection fraction was over 75%. Telemetry showed no arrhythmia. She was scheduled for an outpatient transesophageal echocardiogram, but this was canceled when the patient stated she had been having hematochezia. The hematochezia was found to be secondary to a rectal polyp and resolved after colonoscopy. The transesophageal echocardiogram was never performed.

Syphilis and lyme serologies were negative. PT, PTT, and INR were normal. ESR was 50. Serial troponin values were normal. LDL was 127, HDL was 65, and triglycerides were 75. TSH and B12 were within normal limits. ANA titer was positive at 40 with a diffuse pattern, and the anti-DS DNA test was negative.

Neurology consultation was obtained. The patient was diagnosed with a left INO with left skew deviation secondary to cocaine induced stroke. She was also diagnosed with glaucoma and started on glaucoma medications. Symptoms remained stable during her hospitalization. She was sent to a rehabilitation facility but was lost to follow-up.

## 3. Discussion

Internuclear ophthalmoplegia (INO) is a disorder of conjugate gaze due to a defect in the medial longitudinal fasciculus (MLF). In a literature search for cocaine induced INO, only two case reports and one poster presentation were found. Diaz-Calderon et al. reported a case of cocaine induced hemorrhagic stroke of the midbrain tegmentum, resulting in an INO [1]. Strupp et al. reported a case of an ischemic stroke causing an INO, but this was following coadministration of amphetamine and cocaine [2]. Their case demonstrated a single unilateral T2 intense area near the midline of the mesencephalon. In contrast, our patient suffered multiple ischemic infarctions of the pontine microvasculature as well as an ischemic infarction of the left cerebellum. We believe that one of the pontine lesions was responsible for the INO.

The MLF is necessary for horizontal conjugate gaze. For conjugate horizontal eye movements to occur, motor neuron axons from the abducens nucleus innervate the ipsilateral lateral rectus muscle, causing abduction. The internuclear neurons from the abducens nucleus (the MLF) cross the midline and ascend to the contralateral CN III medial rectus subnucleus activating the medial rectus on that side, causing adduction. In MLF injury, the affected eye (ipsilateral to the site of injury) has an adduction deficit while the contralateral eye demonstrates abducting nystagmus with fast phase towards the abducting direction [7]. In our case, the left hypertropia was due to a skew deviation. Skew deviation frequently occurs as a hypertropia on the side of the INO. Skew deviation is known to occur with strokes of the brainstem or cerebellum [8]. Exotropia in primary gaze is more typical of bilateral INO; however, it may occur in unilateral INO as was seen in our patient [8, 9].

Multiple factors likely contribute to cocaine induced stroke. Cocaine is a sympathomimetic drug that prevents the reuptake of norepinephrine, dopamine, and serotonin. The surge of these neurotransmitters causes tachycardia, vasoconstriction, hypertension, and cerebral vasospasm. Cocaine may also induce a vasculitis [3–6, 10]. The cause of cocaine induced vasculitis is unknown, but cocaine has been shown to induce apoptosis in cerebrovascular smooth muscle cells and to increase leukocyte migration across cerebral blood vessel walls [10]. Because cocaine has a half-life of only one hour, one may expect its effects to wear off quickly. However, its breakdown products can induce vasoconstriction and vasospasm for days [4]. These effects last longer in chronic abusers [11, 12].

There is conflicting evidence as to whether or not cocaine induces or inhibits platelet aggregation. Togna et al. showed that platelet responsiveness varied depending on cocaine concentration and whether platelets were exposed to arachidonic acid, collagen, or ADP and collagen. Some combinations were activating while others were inhibitory [13]. Kugelmass et al. concluded that, in vitro, cocaine increased platelet activation, but only to significant levels in fewer than 50 percent of blood donor samples [14]. In a second article, Kugelmass et al. demonstrated in vivo that platelet activation increased in dogs upon exposure to cocaine [15]. Rinder et al. measured levels of activated platelets in current cocaine users and showed that only a small portion of samples had increased platelet activation. They also showed that in vitro exposure of blood to concentrations of cocaine documented as achievable in vivo had no effect on platelet aggregation [16]. Jennings et al. exposed blood in vitro to cocaine and concluded that cocaine negatively effects platelet aggregation and dissociates preformed platelet aggregates [17]. There is no clear explanation for these disparate findings.

Our patient's presentation of cocaine induced INO due to ischemic stroke is an infrequent occurrence. A similar case may present to a neurologist, an ophthalmologist, or an emergency medicine physician. Common causes of INO include stroke, multiple sclerosis, and tumor; however, as with any oculomotor disturbance, it is important to inquire about recreational drug use [18, 19].

## Supplementary Material

Supplementary Video: This video demonstrates our patient's extraocular movements. Note the left INO, the left skew deviation, and the right abducting nystagmus on attempted right gaze.
